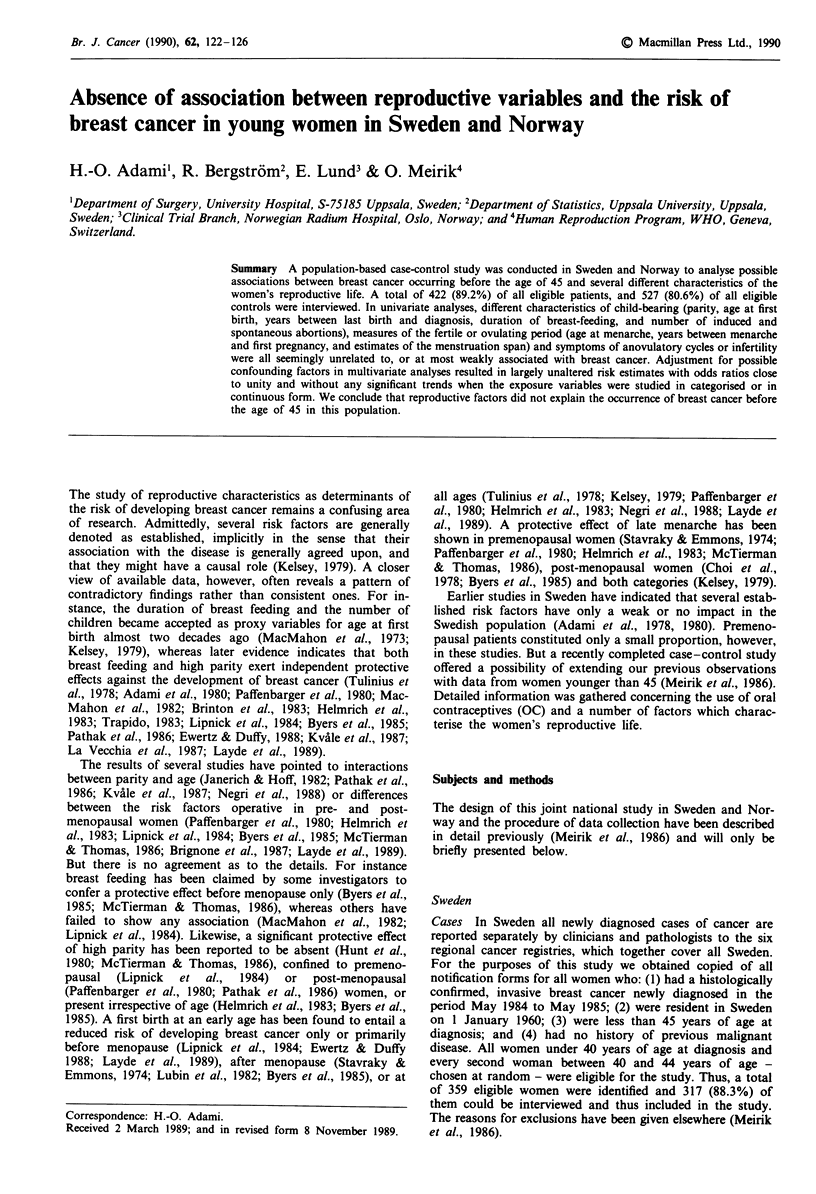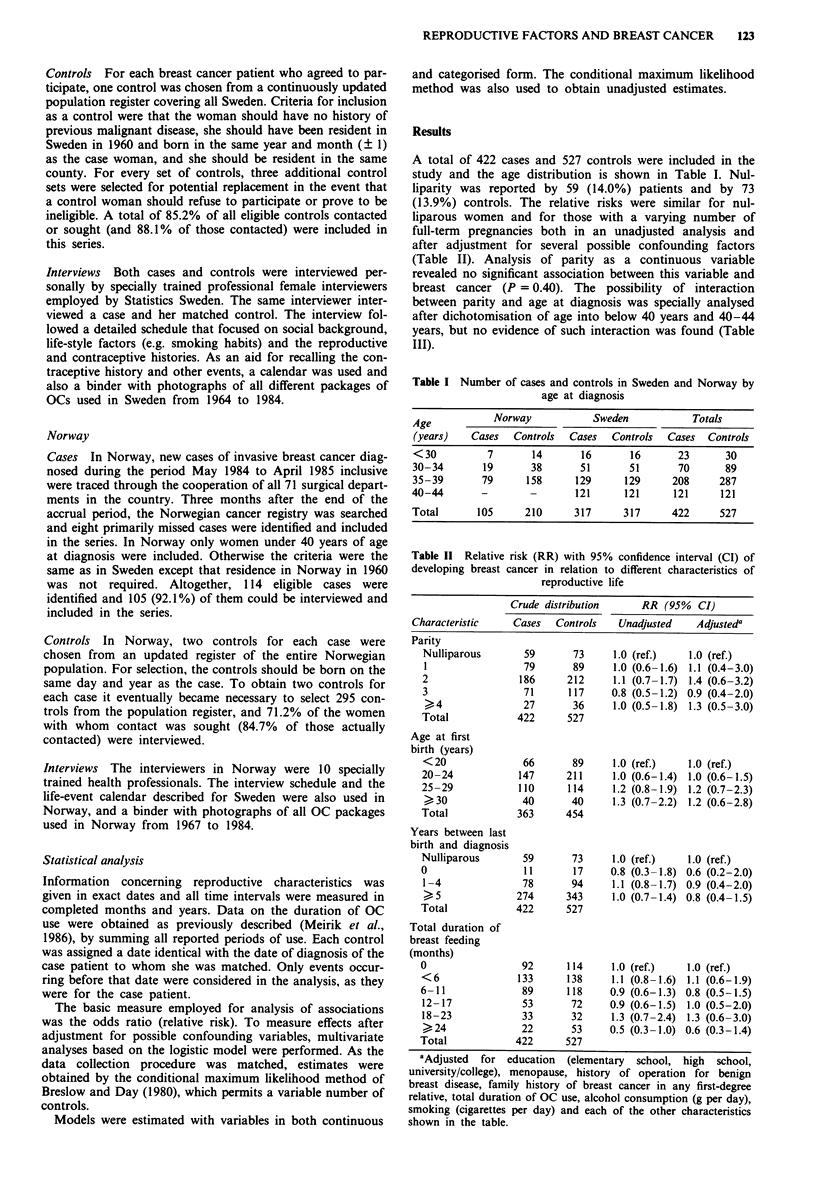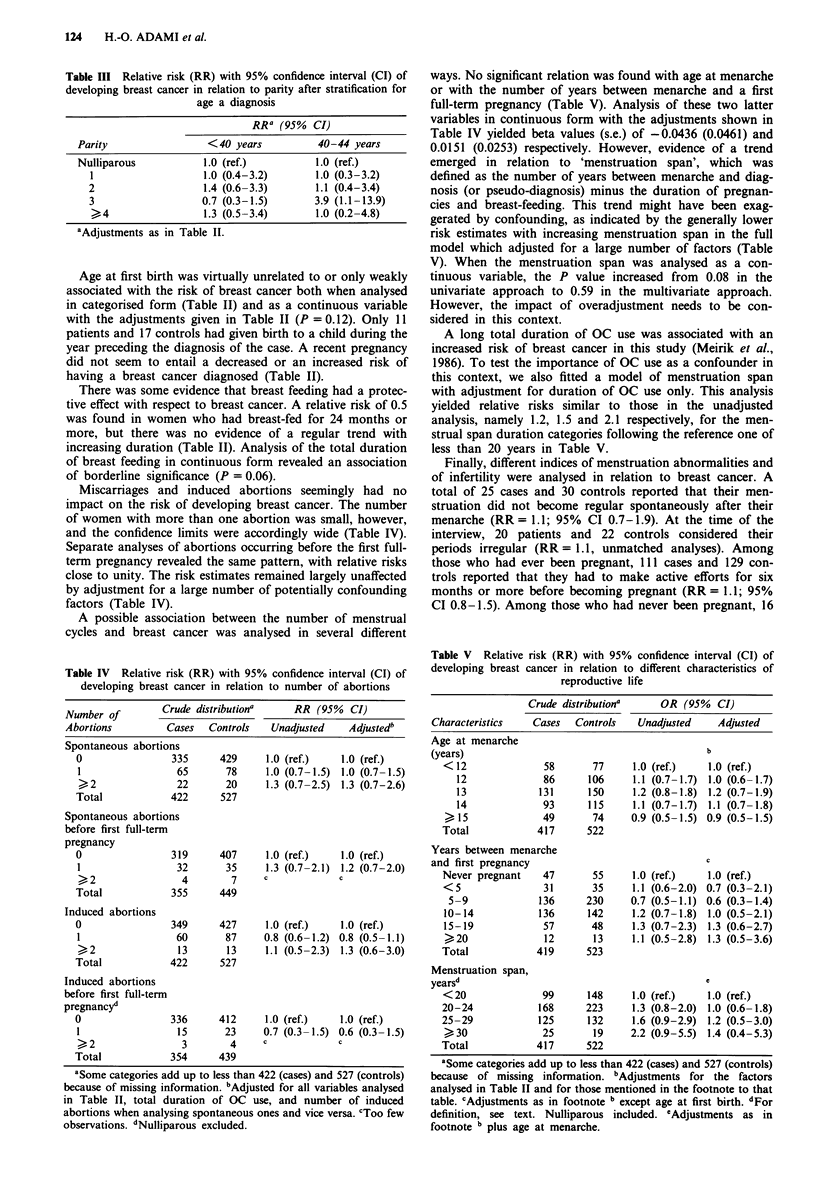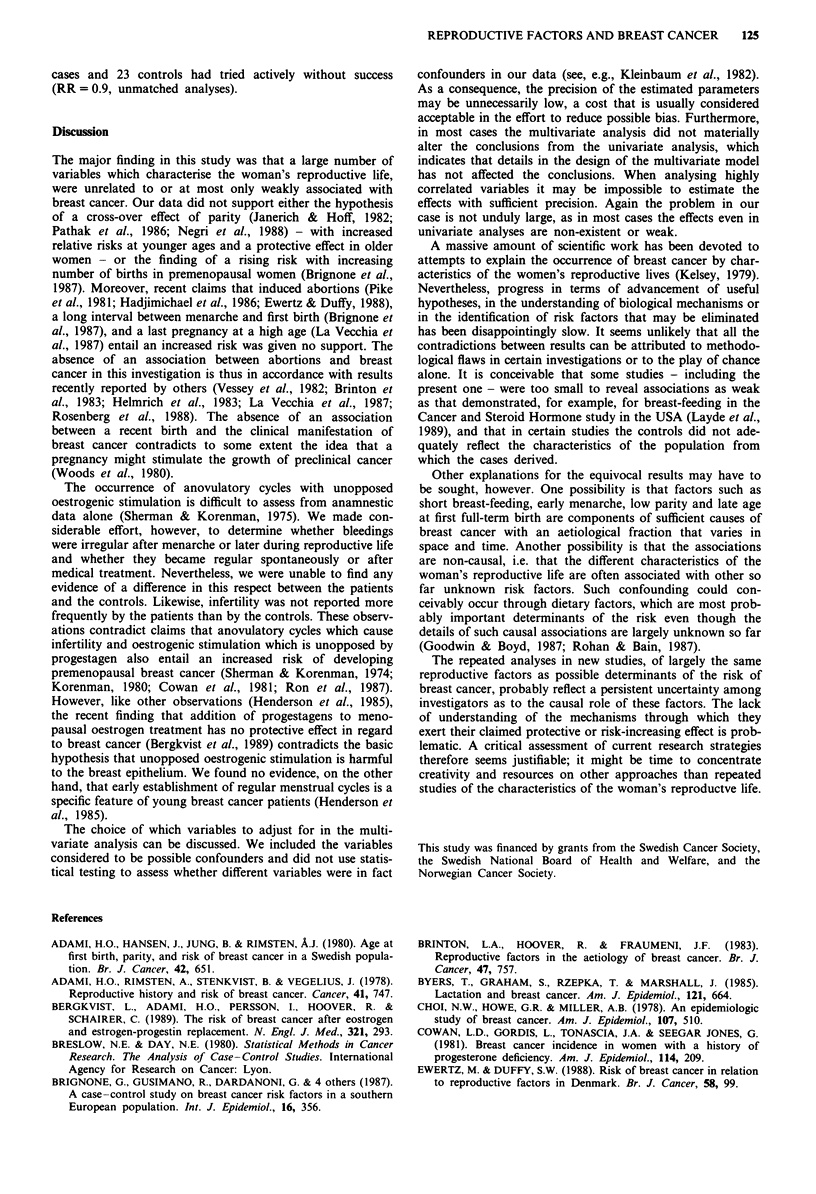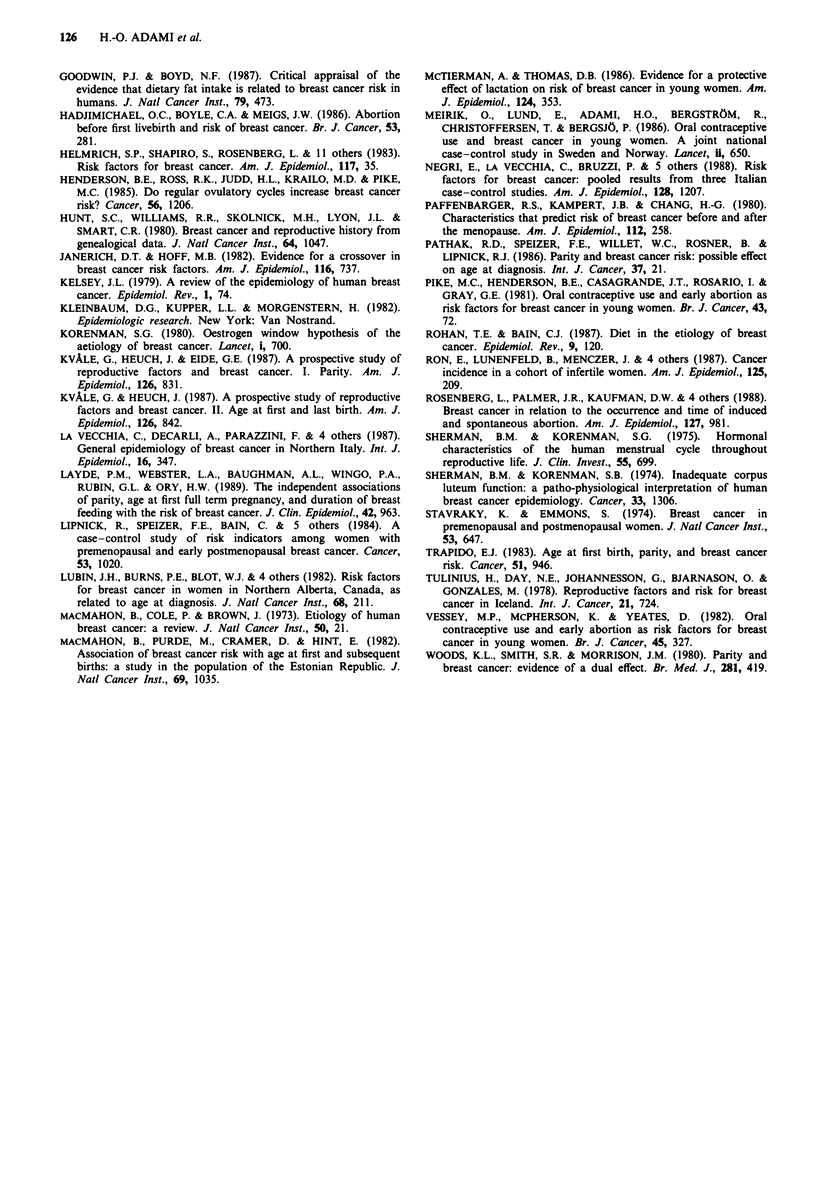# Absence of association between reproductive variables and the risk of breast cancer in young women in Sweden and Norway.

**DOI:** 10.1038/bjc.1990.242

**Published:** 1990-07

**Authors:** H. O. Adami, R. Bergström, E. Lund, O. Meirik

**Affiliations:** Department of Surgery, University Hospital, Uppsala, Sweden.

## Abstract

A population-based case-control study was conducted in Sweden and Norway to analyse possible associations between breast cancer occurring before the age of 45 and several different characteristics of the women's reproductive life. A total of 422 (89.2%) of all eligible patients, and 527 (80.6%) of all eligible controls were interviewed. In univariate analyses, different characteristics of child-bearing (parity, age at first birth, years between last birth and diagnosis, duration of breast-feeding, and number of induced and spontaneous abortions), measures of the fertile or ovulating period (age at menarche, years between menarche and first pregnancy, and estimates of the menstruation span) and symptoms of anovulatory cycles or infertility were all seemingly unrelated to, or at most weakly associated with breast cancer. Adjustment for possible confounding factors in multivariate analyses resulted in largely unaltered risk estimates with odds ratios close to unity and without any significant trends when the exposure variables were studied in categorised or in continuous form. We conclude that reproductive factors did not explain the occurrence of breast cancer before the age of 45 in this population.


Br. J. Cancer (1990), 62, 122-126 © Macmillan Press Ltd., 1990

### Absence of association between reproductive variables and the risk of breast cancer in young women in Sweden and Norway

H.-O. Adami[1], R. Bergström[2], E. Lund[3] & O. Meirik[4]

[1]*Department of Surgery, University Hospital, S-75185 Uppsala, Sweden;* [2]*Department of Statistics, Uppsala University, Uppsala, Sweden;* [3]*Clinical Trial Branch, Norwegian Radium Hospital, Oslo, Norway; and* [4]*Human Reproduction Program, WHO, Geneva, Switzerland.*

**Summary** A population-based case-control study was conducted in Sweden and Norway to analyse possible associations between breast cancer occurring before the age of 45 and several different characteristics of the women's reproductive life. A total of 422 (89.2%) of all eligible patients, and 527 (80.6%) of all eligible controls were interviewed. In univariate analyses, different characteristics of child-bearing (parity, age at first birth, years between last birth and diagnosis, duration of breast-feeding, and number of induced and spontaneous abortions), measures of the fertile or ovulating period (age at menarche, years between menarche and first pregnancy, and estimates of the menstruation span) and symptoms of anovulatory cycles or infertility were all seemingly unrelated to, or at most weakly associated with breast cancer. Adjustment for possible confounding factors in multivariate analyses resulted in largely unaltered risk estimates with odds ratios close to unity and without any significant trends when the exposure variables were studied in categorised or in continuous form. We conclude that reproductive factors did not explain the occurrence of breast cancer before the age of 45 in this population.

The study of reproductive characteristics as determinants of the risk of developing breast cancer remains a confusing area of research. Admittedly, several risk factors are generally denoted as established, implicitly in the sense that their association with the disease is generally agreed upon, and that they might have a causal role (Kelsey, 1979). A closer view of available data, however, often reveals a pattern of contradictory findings rather than consistent ones. For instance, the duration of breast feeding and the number of children became accepted as proxy variables for age at first birth almost two decades ago (MacMahon *et al.*, 1973; Kelsey, 1979), whereas later evidence indicates that both breast feeding and high parity exert independent protective effects against the development of breast cancer (Tulinius *et al.*, 1978; Adami *et al.*, 1980; Paffenbarger *et al.*, 1980; MacMahon *et al.*, 1982; Brinton *et al.*, 1983; Helmrich *et al.*, 1983; Trapido, 1983; Lipnick *et al.*, 1984; Byers *et al.*, 1985; Pathak *et al.*, 1986; Ewertz & Duffy, 1988; Kvåle *et al.*, 1987; La Vecchia *et al.*, 1987; Layde *et al.*, 1989).

The results of several studies have pointed to interactions between parity and age (Janerich & Hoff, 1982; Pathak *et al.*, 1986; Kvåle *et al.*, 1987; Negri *et al.*, 1988) or differences between the risk factors operative in pre- and post-menopausal women (Paffenbarger *et al.*, 1980; Helmrich *et al.*, 1983; Lipnick *et al.*, 1984; Byers *et al.*, 1985; McTierman & Thomas, 1986; Brignone *et al.*, 1987; Layde *et al.*, 1989). But there is no agreement as to the details. For instance breast feeding has been claimed by some investigators to confer a protective effect before menopause only (Byers *et al.*, 1985; McTierman & Thomas, 1986), whereas others have failed to show any association (MacMahon *et al.*, 1982; Lipnick *et al.*, 1984). Likewise, a significant protective effect of high parity has been reported to be absent (Hunt *et al.*, 1980; McTierman & Thomas, 1986), confined to premenopausal (Lipnick *et al.*, 1984) or post-menopausal (Paffenbarger *et al.*, 1980; Pathak *et al.*, 1986) women, or present irrespective of age (Helmrich *et al.*, 1983; Byers *et al.*, 1985). A first birth at an early age has been found to entail a reduced risk of developing breast cancer only or primarily before menopause (Lipnick *et al.*, 1984; Ewertz & Duffy 1988; Layde *et al.*, 1989), after menopause (Stavraky & Emmons, 1974; Lubin *et al.*, 1982; Byers *et al.*, 1985), or at all ages (Tulinius *et al.*, 1978; Kelsey, 1979; Paffenbarger *et al.*, 1980; Helmrich *et al.*, 1983; Negri *et al.*, 1988; Layde *et al.*, 1989). A protective effect of late menarche has been shown in premenopausal women (Stavraky & Emmons, 1974; Paffenbarger *et al.*, 1980; Helmrich *et al.*, 1983; McTierman & Thomas, 1986), post-menopausal women (Choi *et al.*, 1978; Byers *et al.*, 1985) and both categories (Kelsey, 1979).

Earlier studies in Sweden have indicated that several established risk factors have only a weak or no impact in the Swedish population (Adami *et al.*, 1978, 1980). Premenopausal patients constituted only a small proportion, however, in these studies. But a recently completed case-control study offered a possibility of extending our previous observations with data from women younger than 45 (Meirik *et al.*, 1986). Detailed information was gathered concerning the use of oral contraceptives (OC) and a number of factors which characterise the women's reproductive life.

### Subjects and methods

The design of this joint national study in Sweden and Norway and the procedure of data collection have been described in detail previously (Meirik *et al.*, 1986) and will only be briefly presented below.

### *Sweden*

*Cases* In Sweden all newly diagnosed cases of cancer are reported separately by clinicians and pathologists to the six regional cancer registries, which together cover all Sweden. For the purposes of this study we obtained copied of all notification forms for all women who: (1) had a histologically confirmed, invasive breast cancer newly diagnosed in the period May 1984 to May 1985; (2) were resident in Sweden on 1 January 1960; (3) were less than 45 years of age at diagnosis; and (4) had no history of previous malignant disease. All women under 40 years of age at diagnosis and every second woman between 40 and 44 years of age – chosen at random – were eligible for the study. Thus, a total of 359 eligible women were identified and 317 (88.3%) of them could be interviewed and thus included in the study. The reasons for exclusions have been given elsewhere (Meirik *et al.*, 1986).

Correspondence: H.-O. Adami.
Received 2 March 1989; and in revised form 8 November 1989.

*Controls* For each breast cancer patient who agreed to participate, one control was chosen from a continuously updated population register covering all Sweden. Criteria for inclusion as a control were that the woman should have no history of previous malignant disease, she should have been resident in Sweden in 1960 and born in the same year and month (± 1) as the case woman, and she should be resident in the same county. For every set of controls, three additional control sets were selected for potential replacement in the event that a control woman should refuse to participate or prove to be ineligible. A total of 85.2% of all eligible controls contacted or sought (and 88.1% of those contacted) were included in this series.

*Interviews* Both cases and controls were interviewed personally by specially trained professional female interviewers employed by Statistics Sweden. The same interviewer interviewed a case and her matched control. The interview followed a detailed schedule that focused on social background, life-style factors (e.g. smoking habits) and the reproductive and contraceptive histories. As an aid for recalling the contraceptive history and other events, a calendar was used and also a binder with photographs of all different packages of OCs used in Sweden from 1964 to 1984.

### *Norway*

*Cases* In Norway, new cases of invasive breast cancer diagnosed during the period May 1984 to April 1985 inclusive were traced through the cooperation of all 71 surgical departments in the country. Three months after the end of the accrual period, the Norwegian cancer registry was searched and eight primarily missed cases were identified and included in the series. In Norway only women under 40 years of age at diagnosis were included. Otherwise the criteria were the same as in Sweden except that residence in Norway in 1960 was not required. Altogether, 114 eligible cases were identified and 105 (92.1%) of them could be interviewed and included in the series.

*Controls* In Norway, two controls for each case were chosen from an updated register of the entire Norwegian population. For selection, the controls should be born on the same day and year as the case. To obtain two controls for each case it eventually became necessary to select 295 controls from the population register, and 71.2% of the women with whom contact was sought (84.7% of those actually contacted) were interviewed.

*Interviews* The interviewers in Norway were 10 specially trained health professionals. The interview schedule and the life-event calendar described for Sweden were also used in Norway, and a binder with photographs of all OC packages used in Norway from 1967 to 1984.

### *Statistical analysis*

Information concerning reproductive characteristics was given in exact dates and all time intervals were measured in completed months and years. Data on the duration of OC use were obtained as previously described (Meirik *et al.*, 1986), by summing all reported periods of use. Each control was assigned a date identical with the date of diagnosis of the case patient to whom she was matched. Only events occurring before that date were considered in the analysis, as they were for the case patient.

The basic measure employed for analysis of associations was the odds ratio (relative risk). To measure effects after adjustment for possible confounding variables, multivariate analyses based on the logistic model were performed. As the data collection procedure was matched, estimates were obtained by the conditional maximum likelihood method of Breslow and Day (1980), which permits a variable number of controls.

Models were estimated with variables in both continuous and categorised form. The conditional maximum likelihood method was also used to obtain unadjusted estimates.

### Results

A total of 422 cases and 527 controls were included in the study and the age distribution is shown in Table I. Nulliparity was reported by 59 (14.0%) patients and by 73 (13.9%) controls. The relative risks were similar for nulliparous women and for those with a varying number of full-term pregnancies both in an unadjusted analysis and after adjustment for several possible confounding factors (Table II). Analysis of parity as a continuous variable revealed no significant association between this variable and breast cancer ($P = 0.40$). The possibility of interaction between parity and age at diagnosis was specially analysed after dichotomisation of age into below 40 years and 40–44 years, but no evidence of such interaction was found (Table III).

**Table I** Number of cases and controls in Sweden and Norway by age at diagnosis

| *Age (years)* | *Norway* | | *Sweden* | | *Totals* | |
|---|---|---|---|---|---|---|
| | *Cases* | *Controls* | *Cases* | *Controls* | *Cases* | *Controls* |
| <30 | 7 | 14 | 16 | 16 | 23 | 30 |
| 30–34 | 19 | 38 | 51 | 51 | 70 | 89 |
| 35–39 | 79 | 158 | 129 | 129 | 208 | 287 |
| 40–44 | – | – | 121 | 121 | 121 | 121 |
| Total | 105 | 210 | 317 | 317 | 422 | 527 |

**Table II** Relative risk (RR) with 95% confidence interval (CI) of developing breast cancer in relation to different characteristics of reproductive life

| | *Crude distribution* | | *RR (95% CI)* | |
|---|---|---|---|---|
| *Characteristic* | *Cases* | *Controls* | *Unadjusted* | *Adjusted*[a] |
| Parity | | | | |
| Nulliparous | 59 | 73 | 1.0 (ref.) | 1.0 (ref.) |
| 1 | 79 | 89 | 1.0 (0.6–1.6) | 1.1 (0.4–3.0) |
| 2 | 186 | 212 | 1.1 (0.7–1.7) | 1.4 (0.6–3.2) |
| 3 | 71 | 117 | 0.8 (0.5–1.2) | 0.9 (0.4–2.0) |
| ⩾4 | 27 | 36 | 1.0 (0.5–1.8) | 1.3 (0.5–3.0) |
| Total | 422 | 527 | | |
| Age at first birth (years) | | | | |
| <20 | 66 | 89 | 1.0 (ref.) | 1.0 (ref.) |
| 20–24 | 147 | 211 | 1.0 (0.6–1.4) | 1.0 (0.6–1.5) |
| 25–29 | 110 | 114 | 1.2 (0.8–1.9) | 1.2 (0.7–2.3) |
| ⩾30 | 40 | 40 | 1.3 (0.7–2.2) | 1.2 (0.6–2.8) |
| Total | 363 | 454 | | |
| Years between last birth and diagnosis | | | | |
| Nulliparous | 59 | 73 | 1.0 (ref.) | 1.0 (ref.) |
| 0 | 11 | 17 | 0.8 (0.3–1.8) | 0.6 (0.2–2.0) |
| 1–4 | 78 | 94 | 1.1 (0.8–1.7) | 0.9 (0.4–2.0) |
| ⩾5 | 274 | 343 | 1.0 (0.7–1.4) | 0.8 (0.4–1.5) |
| Total | 422 | 527 | | |
| Total duration of breast feeding (months) | | | | |
| 0 | 92 | 114 | 1.0 (ref.) | 1.0 (ref.) |
| <6 | 133 | 138 | 1.1 (0.8–1.6) | 1.1 (0.6–1.9) |
| 6–11 | 89 | 118 | 0.9 (0.6–1.3) | 0.8 (0.5–1.5) |
| 12–17 | 53 | 72 | 0.9 (0.6–1.5) | 1.0 (0.5–2.0) |
| 18–23 | 33 | 32 | 1.3 (0.7–2.4) | 1.3 (0.6–3.0) |
| ⩾24 | 22 | 53 | 0.5 (0.3–1.0) | 0.6 (0.3–1.4) |
| Total | 422 | 527 | | |

[a]Adjusted for education (elementary school, high school, university/college), menopause, history of operation for benign breast disease, family history of breast cancer in any first-degree relative, total duration of OC use, alcohol consumption (g per day), smoking (cigarettes per day) and each of the other characteristics shown in the table.

**Table III** Relative risk (RR) with 95% confidence interval (CI) of developing breast cancer in relation to parity after stratification for age a diagnosis

| Parity | RR[a] (95% CI) <40 years | RR[a] (95% CI) 40-44 years |
|---|---|---|
| Nulliparous | 1.0 (ref.) | 1.0 (ref.) |
| 1 | 1.0 (0.4-3.2) | 1.0 (0.3-3.2) |
| 2 | 1.4 (0.6-3.3) | 1.1 (0.4-3.4) |
| 3 | 0.7 (0.3-1.5) | 3.9 (1.1-13.9) |
| ≥4 | 1.3 (0.5-3.4) | 1.0 (0.2-4.8) |

[a]Adjustments as in Table II.

Age at first birth was virtually unrelated to or only weakly associated with the risk of breast cancer both when analysed in categorised form (Table II) and as a continuous variable with the adjustments given in Table II ($P = 0.12$). Only 11 patients and 17 controls had given birth to a child during the year preceding the diagnosis of the case. A recent pregnancy did not seem to entail a decreased or an increased risk of having a breast cancer diagnosed (Table II).

There was some evidence that breast feeding had a protective effect with respect to breast cancer. A relative risk of 0.5 was found in women who had breast-fed for 24 months or more, but there was no evidence of a regular trend with increasing duration (Table II). Analysis of the total duration of breast feeding in continuous form revealed an association of borderline significance ($P = 0.06$).

Miscarriages and induced abortions seemingly had no impact on the risk of developing breast cancer. The number of women with more than one abortion was small, however, and the confidence limits were accordingly wide (Table IV). Separate analyses of abortions occurring before the first full-term pregnancy revealed the same pattern, with relative risks close to unity. The risk estimates remained largely unaffected by adjustment for a large number of potentially confounding factors (Table IV).

A possible association between the number of menstrual cycles and breast cancer was analysed in several different ways. No significant relation was found with age at menarche or with the number of years between menarche and a first full-term pregnancy (Table V). Analysis of these two latter variables in continuous form with the adjustments shown in Table IV yielded beta values (s.e.) of −0.0436 (0.0461) and 0.0151 (0.0253) respectively. However, evidence of a trend emerged in relation to 'menstruation span', which was defined as the number of years between menarche and diagnosis (or pseudo-diagnosis) minus the duration of pregnancies and breast-feeding. This trend might have been exaggerated by confounding, as indicated by the generally lower risk estimates with increasing menstruation span in the full model which adjusted for a large number of factors (Table V). When the menstruation span was analysed as a continuous variable, the $P$ value increased from 0.08 in the univariate approach to 0.59 in the multivariate approach. However, the impact of overadjustment needs to be considered in this context.

A long total duration of OC use was associated with an increased risk of breast cancer in this study (Meirik et al., 1986). To test the importance of OC use as a confounder in this context, we also fitted a model of menstruation span with adjustment for duration of OC use only. This analysis yielded relative risks similar to those in the unadjusted analysis, namely 1.2, 1.5 and 2.1 respectively, for the menstrual span duration categories following the reference one of less than 20 years in Table V.

Finally, different indices of menstruation abnormalities and of infertility were analysed in relation to breast cancer. A total of 25 cases and 30 controls reported that their menstruation did not become regular spontaneously after their menarche (RR = 1.1; 95% CI 0.7-1.9). At the time of the interview, 20 patients and 22 controls considered their periods irregular (RR = 1.1, unmatched analyses). Among those who had ever been pregnant, 111 cases and 129 controls reported that they had to make active efforts for six months or more before becoming pregnant (RR = 1.1; 95% CI 0.8-1.5). Among those who had never been pregnant, 16

**Table IV** Relative risk (RR) with 95% confidence interval (CI) of developing breast cancer in relation to number of abortions

| Number of Abortions | Crude distribution[a] Cases | Crude distribution[a] Controls | RR (95% CI) Unadjusted | RR (95% CI) Adjusted[b] |
|---|---|---|---|---|
| Spontaneous abortions | | | | |
| 0 | 335 | 429 | 1.0 (ref.) | 1.0 (ref.) |
| 1 | 65 | 78 | 1.0 (0.7-1.5) | 1.0 (0.7-1.5) |
| ≥2 | 22 | 20 | 1.3 (0.7-2.5) | 1.3 (0.7-2.6) |
| Total | 422 | 527 | | |
| Spontaneous abortions before first full-term pregnancy | | | | |
| 0 | 319 | 407 | 1.0 (ref.) | 1.0 (ref.) |
| 1 | 32 | 35 | 1.3 (0.7-2.1) | 1.2 (0.7-2.0) |
| ≥2 | 4 | 7 | c | c |
| Total | 355 | 449 | | |
| Induced abortions | | | | |
| 0 | 349 | 427 | 1.0 (ref.) | 1.0 (ref.) |
| 1 | 60 | 87 | 0.8 (0.6-1.2) | 0.8 (0.5-1.1) |
| ≥2 | 13 | 13 | 1.1 (0.5-2.3) | 1.3 (0.6-3.0) |
| Total | 422 | 527 | | |
| Induced abortions before first full-term pregnancy[d] | | | | |
| 0 | 336 | 412 | 1.0 (ref.) | 1.0 (ref.) |
| 1 | 15 | 23 | 0.7 (0.3-1.5) | 0.6 (0.3-1.5) |
| ≥2 | 3 | 4 | c | c |
| Total | 354 | 439 | | |

[a]Some categories add up to less than 422 (cases) and 527 (controls) because of missing information. [b]Adjusted for all variables analysed in Table II, total duration of OC use, and number of induced abortions when analysing spontaneous ones and vice versa. [c]Too few observations. [d]Nulliparous excluded.

**Table V** Relative risk (RR) with 95% confidence interval (CI) of developing breast cancer in relation to different characteristics of reproductive life

| Characteristics | Crude distribution[a] Cases | Crude distribution[a] Controls | OR (95% CI) Unadjusted | OR (95% CI) Adjusted |
|---|---|---|---|---|
| Age at menarche (years) | | | | b |
| <12 | 58 | 77 | 1.0 (ref.) | 1.0 (ref.) |
| 12 | 86 | 106 | 1.1 (0.7-1.7) | 1.0 (0.6-1.7) |
| 13 | 131 | 150 | 1.2 (0.8-1.8) | 1.2 (0.7-1.9) |
| 14 | 93 | 115 | 1.1 (0.7-1.7) | 1.1 (0.7-1.8) |
| ≥15 | 49 | 74 | 0.9 (0.5-1.5) | 0.9 (0.5-1.5) |
| Total | 417 | 522 | | |
| Years between menarche and first pregnancy | | | | c |
| Never pregnant | 47 | 55 | 1.0 (ref.) | 1.0 (ref.) |
| <5 | 31 | 35 | 1.1 (0.6-2.0) | 0.7 (0.3-2.1) |
| 5-9 | 136 | 230 | 0.7 (0.5-1.1) | 0.6 (0.3-1.4) |
| 10-14 | 136 | 142 | 1.2 (0.7-1.8) | 1.0 (0.5-2.1) |
| 15-19 | 57 | 48 | 1.3 (0.7-2.3) | 1.3 (0.6-2.7) |
| ≥20 | 12 | 13 | 1.1 (0.5-2.8) | 1.3 (0.5-3.6) |
| Total | 419 | 523 | | |
| Menstruation span, years[d] | | | | e |
| <20 | 99 | 148 | 1.0 (ref.) | 1.0 (ref.) |
| 20-24 | 168 | 223 | 1.3 (0.8-2.0) | 1.0 (0.6-1.8) |
| 25-29 | 125 | 132 | 1.6 (0.9-2.9) | 1.2 (0.5-3.0) |
| ≥30 | 25 | 19 | 2.2 (0.9-5.5) | 1.4 (0.4-5.3) |
| Total | 417 | 522 | | |

[a]Some categories add up to less than 422 (cases) and 527 (controls) because of missing information. [b]Adjustments for the factors analysed in Table II and for those mentioned in the footnote to that table. [c]Adjustments as in footnote [b] except age at first birth. [d]For definition, see text. Nulliparous included. [e]Adjustments as in footnote [b] plus age at menarche.

cases and 23 controls had tried actively without success (RR = 0.9, unmatched analyses).

### Discussion

The major finding in this study was that a large number of variables which characterise the woman's reproductive life, were unrelated to or at most only weakly associated with breast cancer. Our data did not support either the hypothesis of a cross-over effect of parity (Janerich & Hoff, 1982; Pathak *et al.*, 1986; Negri *et al.*, 1988) – with increased relative risks at younger ages and a protective effect in older women – or the finding of a rising risk with increasing number of births in premenopausal women (Brignone *et al.*, 1987). Moreover, recent claims that induced abortions (Pike *et al.*, 1981; Hadjimichael *et al.*, 1986; Ewertz & Duffy, 1988), a long interval between menarche and first birth (Brignone *et al.*, 1987), and a last pregnancy at a high age (La Vecchia *et al.*, 1987) entail an increased risk was given no support. The absence of an association between abortions and breast cancer in this investigation is thus in accordance with results recently reported by others (Vessey *et al.*, 1982; Brinton *et al.*, 1983; Helmrich *et al.*, 1983; La Vecchia *et al.*, 1987; Rosenberg *et al.*, 1988). The absence of an association between a recent birth and the clinical manifestation of breast cancer contradicts to some extent the idea that a pregnancy might stimulate the growth of preclinical cancer (Woods *et al.*, 1980).

The occurrence of anovulatory cycles with unopposed oestrogenic stimulation is difficult to assess from anamnestic data alone (Sherman & Korenman, 1975). We made considerable effort, however, to determine whether bleedings were irregular after menarche or later during reproductive life and whether they became regular spontaneously or after medical treatment. Nevertheless, we were unable to find any evidence of a difference in this respect between the patients and the controls. Likewise, infertility was not reported more frequently by the patients than by the controls. These observations contradict claims that anovulatory cycles which cause infertility and oestrogenic stimulation which is unopposed by progestagen also entail an increased risk of developing premenopausal breast cancer (Sherman & Korenman, 1974; Korenman, 1980; Cowan *et al.*, 1981; Ron *et al.*, 1987). However, like other observations (Henderson *et al.*, 1985), the recent finding that addition of progestagens to menopausal oestrogen treatment has no protective effect in regard to breast cancer (Bergkvist *et al.*, 1989) contradicts the basic hypothesis that unopposed oestrogenic stimulation is harmful to the breast epithelium. We found no evidence, on the other hand, that early establishment of regular menstrual cycles is a specific feature of young breast cancer patients (Henderson *et al.*, 1985).

The choice of which variables to adjust for in the multivariate analysis can be discussed. We included the variables considered to be possible confounders and did not use statistical testing to assess whether different variables were in fact confounders in our data (see, e.g., Kleinbaum *et al.*, 1982). As a consequence, the precision of the estimated parameters may be unnecessarily low, a cost that is usually considered acceptable in the effort to reduce possible bias. Furthermore, in most cases the multivariate analysis did not materially alter the conclusions from the univariate analysis, which indicates that details in the design of the multivariate model has not affected the conclusions. When analysing highly correlated variables it may be impossible to estimate the effects with sufficient precision. Again the problem in our case is not unduly large, as in most cases the effects even in univariate analyses are non-existent or weak.

A massive amount of scientific work has been devoted to attempts to explain the occurrence of breast cancer by characteristics of the women's reproductive lives (Kelsey, 1979). Nevertheless, progress in terms of advancement of useful hypotheses, in the understanding of biological mechanisms or in the identification of risk factors that may be eliminated has been disappointingly slow. It seems unlikely that all the contradictions between results can be attributed to methodological flaws in certain investigations or to the play of chance alone. It is conceivable that some studies – including the present one – were too small to reveal associations as weak as that demonstrated, for example, for breast-feeding in the Cancer and Steroid Hormone study in the USA (Layde *et al.*, 1989), and that in certain studies the controls did not adequately reflect the characteristics of the population from which the cases derived.

Other explanations for the equivocal results may have to be sought, however. One possibility is that factors such as short breast-feeding, early menarche, low parity and late age at first full-term birth are components of sufficient causes of breast cancer with an aetiological fraction that varies in space and time. Another possibility is that the associations are non-causal, i.e. that the different characteristics of the woman's reproductive life are often associated with other so far unknown risk factors. Such confounding could conceivably occur through dietary factors, which are most probably important determinants of the risk even though the details of such causal associations are largely unknown so far (Goodwin & Boyd, 1987; Rohan & Bain, 1987).

The repeated analyses in new studies, of largely the same reproductive factors as possible determinants of the risk of breast cancer, probably reflect a persistent uncertainty among investigators as to the causal role of these factors. The lack of understanding of the mechanisms through which they exert their claimed protective or risk-increasing effect is problematic. A critical assessment of current research strategies therefore seems justifiable; it might be time to concentrate creativity and resources on other approaches than repeated studies of the characteristics of the woman's reproductve life.

This study was financed by grants from the Swedish Cancer Society, the Swedish National Board of Health and Welfare, and the Norwegian Cancer Society.

### References

ADAMI, H.O., HANSEN, J., JUNG, B. & RIMSTEN, Å.J. (1980). Age at first birth, parity, and risk of breast cancer in a Swedish population. *Br. J. Cancer*, **42**, 651.

ADAMI, H.O., RIMSTEN, A., STENKVIST, B. & VEGELIUS, J. (1978). Reproductive history and risk of breast cancer. *Cancer*, **41**, 747.

BERGKVIST, L., ADAMI, H.O., PERSSON, I., HOOVER, R. & SCHAIRER, C. (1989). The risk of breast cancer after eostrogen and estrogen-progestin replacement. *N. Engl. J. Med.*, **321**, 293.

BRESLOW, N.E. & DAY, N.E. (1980). *Statistical Methods in Cancer Research. The Analysis of Case–Control Studies.* International Agency for Research on Cancer: Lyon.

BRIGNONE, G., GUSIMANO, R., DARDANONI, G. & 4 others (1987). A case–control study on breast cancer risk factors in a southern European population. *Int. J. Epidemiol.*, **16**, 356.

BRINTON, L.A., HOOVER, R. & FRAUMENI, J.F. (1983). Reproductive factors in the aetiology of breast cancer. *Br. J. Cancer*, **47**, 757.

BYERS, T., GRAHAM, S., RZEPKA, T. & MARSHALL, J. (1985). Lactation and breast cancer. *Am. J. Epidemiol.*, **121**, 664.

CHOI, N.W., HOWE, G.R. & MILLER, A.B. (1978). An epidemiologic study of breast cancer. *Am. J. Epidemiol.*, **107**, 510.

COWAN, L.D., GORDIS, L., TONASCIA, J.A. & SEEGAR JONES, G. (1981). Breast cancer incidence in women with a history of progesterone deficiency. *Am. J. Epidemiol.*, **114**, 209.

EWERTZ, M. & DUFFY, S.W. (1988). Risk of breast cancer in relation to reproductive factors in Denmark. *Br. J. Cancer*, **58**, 99.

GOODWIN, P.J. & BOYD, N.F. (1987). Critical appraisal of the evidence that dietary fat intake is related to breast cancer risk in humans. *J. Natl Cancer Inst.*, **79**, 473.

HADJIMICHAEL, O.C., BOYLE, C.A. & MEIGS, J.W. (1986). Abortion before first livebirth and risk of breast cancer. *Br. J. Cancer*, **53**, 281.

HELMRICH, S.P., SHAPIRO, S., ROSENBERG, L. & 11 others (1983). Risk factors for breast cancer. *Am. J. Epidemiol.*, **117**, 35.

HENDERSON, B.E., ROSS, R.K., JUDD, H.L., KRAILO, M.D. & PIKE, M.C. (1985). Do regular ovulatory cycles increase breast cancer risk? *Cancer*, **56**, 1206.

HUNT, S.C., WILLIAMS, R.R., SKOLNICK, M.H., LYON, J.L. & SMART, C.R. (1980). Breast cancer and reproductive history from genealogical data. *J. Natl Cancer Inst.*, **64**, 1047.

JANERICH, D.T. & HOFF, M.B. (1982). Evidence for a crossover in breast cancer risk factors. *Am. J. Epidemiol.*, **116**, 737.

KELSEY, J.L. (1979). A review of the epidemiology of human breast cancer. *Epidemiol. Rev.*, **1**, 74.

KLEINBAUM, D.G., KUPPER, L.L. & MORGENSTERN, H. (1982). *Epidemiologic research*. New York: Van Nostrand.

KORENMAN, S.G. (1980). Oestrogen window hypothesis of the aetiology of breast cancer. *Lancet*, **i**, 700.

KVÅLE, G., HEUCH, J. & EIDE, G.E. (1987). A prospective study of reproductive factors and breast cancer. I. Parity. *Am. J. Epidemiol.*, **126**, 831.

KVÅLE, G. & HEUCH, J. (1987). A prospective study of reproductive factors and breast cancer. II. Age at first and last birth. *Am. J. Epidemiol.*, **126**, 842.

LA VECCHIA, C., DECARLI, A., PARAZZINI, F. & 4 others (1987). General epidemiology of breast cancer in Northern Italy. *Int. J. Epidemiol.*, **16**, 347.

LAYDE, P.M., WEBSTER, L.A., BAUGHMAN, A.L., WINGO, P.A., RUBIN, G.L. & ORY, H.W. (1989). The independent associations of parity, age at first full term pregnancy, and duration of breast feeding with the risk of breast cancer. *J. Clin. Epidemiol.*, **42**, 963.

LIPNICK, R., SPEIZER, F.E., BAIN, C. & 5 others (1984). A case-control study of risk indicators among women with premenopausal and early postmenopausal breast cancer. *Cancer*, **53**, 1020.

LUBIN, J.H., BURNS, P.E., BLOT, W.J. & 4 others (1982). Risk factors for breast cancer in women in Northern Alberta, Canada, as related to age at diagnosis. *J. Natl Cancer Inst.*, **68**, 211.

MACMAHON, B., COLE, P. & BROWN, J. (1973). Etiology of human breast cancer: a review. *J. Natl Cancer Inst.*, **50**, 21.

MACMAHON, B., PURDE, M., CRAMER, D. & HINT, E. (1982). Association of breast cancer risk with age at first and subsequent births: a study in the population of the Estonian Republic. *J. Natl Cancer Inst.*, **69**, 1035.

MCTIERMAN, A. & THOMAS, D.B. (1986). Evidence for a protective effect of lactation on risk of breast cancer in young women. *Am. J. Epidemiol.*, **124**, 353.

MEIRIK, O., LUND, E., ADAMI, H.O., BERGSTRÖM, R., CHRISTOFFERSEN, T. & BERGSJÖ, P. (1986). Oral contraceptive use and breast cancer in young women. A joint national case-control study in Sweden and Norway. *Lancet*, **ii**, 650.

NEGRI, E., LA VECCHIA, C., BRUZZI, P. & 5 others (1988). Risk factors for breast cancer: pooled results from three Italian case-control studies. *Am. J. Epidemiol.*, **128**, 1207.

PAFFENBARGER, R.S., KAMPERT, J.B. & CHANG, H.-G. (1980). Characteristics that predict risk of breast cancer before and after the menopause. *Am. J. Epidemiol.*, **112**, 258.

PATHAK, R.D., SPEIZER, F.E., WILLET, W.C., ROSNER, B. & LIPNICK, R.J. (1986). Parity and breast cancer risk: possible effect on age at diagnosis. *Int. J. Cancer*, **37**, 21.

PIKE, M.C., HENDERSON, B.E., CASAGRANDE, J.T., ROSARIO, I. & GRAY, G.E. (1981). Oral contraceptive use and early abortion as risk factors for breast cancer in young women. *Br. J. Cancer*, **43**, 72.

ROHAN, T.E. & BAIN, C.J. (1987). Diet in the etiology of breast cancer. *Epidemiol. Rev.*, **9**, 120.

RON, E., LUNENFELD, B., MENCZER, J. & 4 others (1987). Cancer incidence in a cohort of infertile women. *Am. J. Epidemiol.*, **125**, 209.

ROSENBERG, L., PALMER, J.R., KAUFMAN, D.W. & 4 others (1988). Breast cancer in relation to the occurrence and time of induced and spontaneous abortion. *Am. J. Epidemiol.*, **127**, 981.

SHERMAN, B.M. & KORENMAN, S.G. (1975). Hormonal characteristics of the human menstrual cycle throughout reproductive life. *J. Clin. Invest.*, **55**, 699.

SHERMAN, B.M. & KORENMAN, S.B. (1974). Inadequate corpus luteum function: a patho-physiological interpretation of human breast cancer epidemiology. *Cancer*, **33**, 1306.

STAVRAKY, K. & EMMONS, S. (1974). Breast cancer in premenopausal and postmenopausal women. *J. Natl Cancer Inst.*, **53**, 647.

TRAPIDO, E.J. (1983). Age at first birth, parity, and breast cancer risk. *Cancer*, **51**, 946.

TULINIUS, H., DAY, N.E., JOHANNESSON, G., BJARNASON, O. & GONZALES, M. (1978). Reproductive factors and risk for breast cancer in Iceland. *Int. J. Cancer*, **21**, 724.

VESSEY, M.P., MCPHERSON, K. & YEATES, D. (1982). Oral contraceptive use and early abortion as risk factors for breast cancer in young women. *Br. J. Cancer*, **45**, 327.

WOODS, K.L., SMITH, S.R. & MORRISON, J.M. (1980). Parity and breast cancer: evidence of a dual effect. *Br. Med. J.*, **281**, 419.